# Tubular adenoma with squamoid morules in colonic polyp: Cases report and literature review

**DOI:** 10.1097/MD.0000000000043965

**Published:** 2025-08-22

**Authors:** Song Iy Han, Sung-Chul Lim

**Affiliations:** a Division of Premedical Science, School of Medicine, Chosun University, Gwangju, Korea; b Department of Pathology, School of Medicine, Chosun University, Gwangju, Korea.

**Keywords:** adenoma, colon, mucin spillage, pseudoinvasion, squamoid morules

## Abstract

**Rationale:**

Squamoid morules (SM) are rare in colorectal adenomas. Submucosal pseudoinvasion in adenomas is similar to that in invasive carcinomas and needs to be differentiated, especially in the presence of mucin spillage.

**Patient concerns:**

We present 2 cases of adenomatous polyps associated with SM. One was approximately 3.2 × 1.8 cm-sized pedunculated polyp in the descending colon of a 57-year-old man, and the other was a 1 cm-sized fungating polyp in the ileocecal valve (ICV) of a 44-year-old man.

**Diagnoses:**

The polyp of the descending colon was accompanied with a low-grade villotubular adenoma with submucosal pseudoinvasion, mucin spillage, and SM; the polyp of the ICV was accompanied with a low-grade tubular adenoma with SM; and transformation to a high-grade tubular adenoma was also observed.

**Interventions:**

The polyps in the descending colon and ICV were removed using endoscopic mucosal resection hot-snare polypectomy and underwater endoscopic mucosal resection, respectively.

**Outcomes:**

The lesions were completely removed, symptomatic improvement was achieved, and no relapse was observed.

**Lessons:**

We encountered 2 cases of SM in adenomas of the colon, one of which was accompanied with submucosal pseudoinvasion and mucin spillage. This represents the 10th reported case of colorectal adenoma with submucosal pseudoinvasion and SM, and the first case report of mucin spillage in addition to submucosal pseudoinvasion and SM. SM are a very rare finding in adenomas and may be difficult to distinguish from malignant lesions with limited sampling, especially when submucosal pseudoinvasion and mucin spillage are additionally present. Available reports should be reviewed to examine the pathogenesis of SM and submucosal pseudoinvasion and to differentiate these from invasive cancer. This will aid in preventing misdiagnosis of invasive carcinoma when such findings occur in benign lesions.

## 1. Introduction

Focal squamous differentiation is rarely observed in colorectal adenoma.^[1–3]^ Electron microscopic research has revealed that these changes are related to squamous metaplasia,^[4]^ and the terms squamous metaplasia, squamoid morules (SM), and squamous differentiation have been used interchangeably.^[1–3,5]^ In 2005, Ueo et al^[6]^ argued that squamous metaplasia and SM should be used differently, but all publications used the same morphological changes observed in colorectal adenoma, without distinguishing between squamous metaplasia, SM, and squamous differentiation.^[1–3,5]^

Invasive growth is a hallmark of cancer, and even in colorectal adenomas, tumors can extend in the submucosa beyond the muscularis mucosa, which is called pseudoinvasion.^[7,8]^ This pseudoinvasion can consist only of neoplastic glands, but sometimes the neoplastic glands can cause distension owing to various degrees of luminal mucin content. Distended glands may cause glandular disruption, resulting in mucin spilling around them. In such cases, pseudoinvasion of the adenoma may be more difficult to differentiate from invasive cancer.^[9,10]^

To date, only 9 cases of colorectal adenoma with submucosal pseudoinvasion and SM have been reported,^[2,9–12]^ but no cases of mucin spillage in addition to submucosal pseudoinvasion and SM have been reported. Submucosal pseudoinvasion with SM is difficult to distinguish from invasive cancer. Limited tissue specimen samples or distortion can lead to overdiagnosis.

We reviewed literature and reported 2 cases to examine the pathogenesis of SM and submucosal pseudoinvasion. Further we revealed important differences between the invasive cancers and SM and pseudoinvasion to aid timely and accurate diagnosis of similar cases.

## 2. Cases report

### 2.1. Case 1

A 57-year-old man reported sighting a trace amount of blood in stool approximately 4 months prior to presentation. He reported spotting a significant amount of blood in stool, experiencing abdominal pain, and visiting a local clinic 3 days prior to presentation. Based on the colonoscopy findings, a large head pedunculated polyp was observed in the descending colon, the patient was transferred to our hospital.

In the colonoscopy findings performed again after transfer, it was judged that the size of the polyp was large, the polyp mucosa was fixed, and there was a possibility of a cancer invading submucosa.

**Figure 1.**
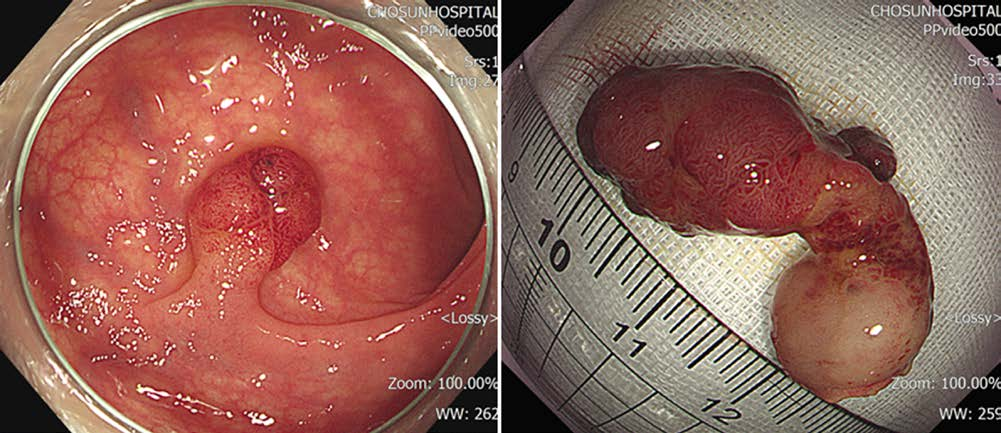
Endoscopic finding. Approximately 3.2 × 1.8 cm sized pedunculated polyp at the descending colon.

The endoscopic diagnosis was submucosal invasive cancer, and endoscopic mucosal resection (EMR) hot-snare polypectomy was performed. The polyp was approximately 3.2 × 1.8 cm in size (Fig. 1).

**Figure 2.**
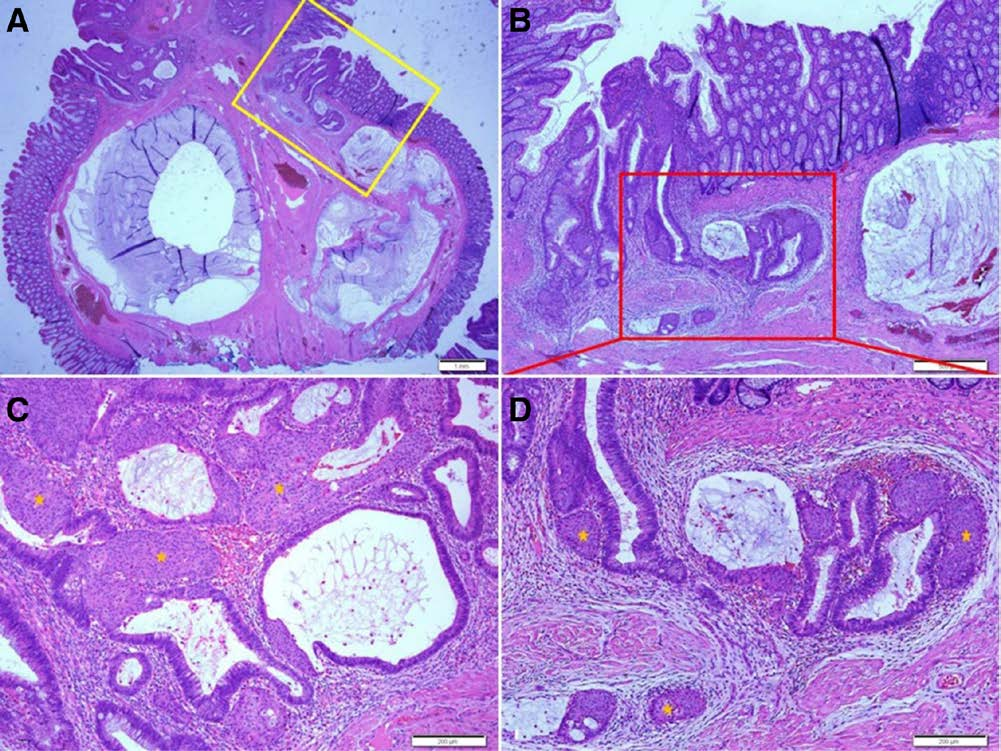
Histopathologic findings of the endoscopic mucosal resection. Low grade villotubular adenoma is identified (A). Higher power view of the yellow boxed area of (A) shows low-grade adenoma with pseudoinvasion and mucin spillage in the submucosa (B). Higher power view of the red boxed area of (B) shows low-grade adenoma with squamoid morules (asterisks) in the pseudoinvasive area. Inflammatory cell infiltration with hemorrhage and myxoid stromal change are observed around the pseudoinvasive lesion without desmoplasia (D). Another focus of low-grade adenoma with squamoid morules (C). Hematoxylin and eosin staining. Scale bars measure, (A) 1 mm, (B) 500 µm, and (C, D) 200 µm.

Histopathological findings of the EMR showed a low-grade villotubular adenoma with pseudoinvasion and mucin spillage in the submucosa. A high-power view showed the SM, mainly around the area where submucosal pseudoinvasion was observed. The tumor glands at the site of pseudoinvasion and mucin spillage showed no obvious cytological atypia or nuclear pleomorphism, and inflammatory cell infiltration with hemorrhage and myxoid stromal changes were observed without desmoplasia around them (Fig. 2).

**Figure 3.**
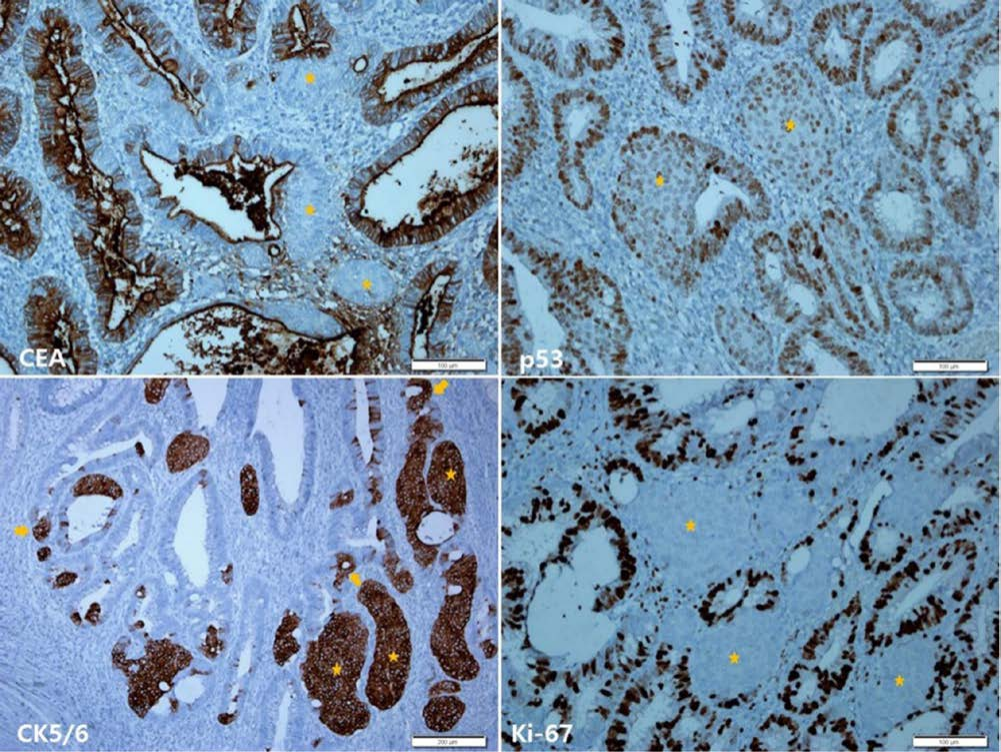
Immunohistochemical findings of the endoscopic mucosal resection. CEA staining shows positive expression in the adenoma, but in the squamoid morules (asterisks; upper left). P53 protein staining shows relatively diffuse strong expression in the adenoma, but weakly scattered expression in the squamoid morule (asterisk; upper right). CK5/6 staining shows diffuse positive expression in the squamoid morules (asterisks) and some non-squamoid glandular epithelia (arrows; lower left). Ki-67 staining shows diffuse positive expression in the adenoma, but negative expression in the squamoid morules (asterisk; lower right). Scale bars measure, CEA, p53, and Ki-67: 100 µm and CK5/6: 200 µm. CEA = carcinoembryonic antigen.

Immunohistochemical findings of the EMR showed carcinoembryonic antigen positivity in the adenoma, but not in the SM. P53 protein staining showed relatively diffuse strong expression in the adenoma, but weakly scattered expression in the SM. Cytokeratin (CK) 5/6 staining showed diffuse positive expression in the SM and some amount of non-squamoid glandular epithelia. Ki-67 staining showed increased expression in the adenoma, but very low expression in the SM (Fig. 3). SM showed negative immunoreactivity for P40, CK20, synaptophysin, chromogranin A, CD56, and insulinoma-associated protein 1.

No signs of recurrence were observed in colonoscopy performed 12 months after EMR.

### 2.2. Case 2

**Figure 4.**
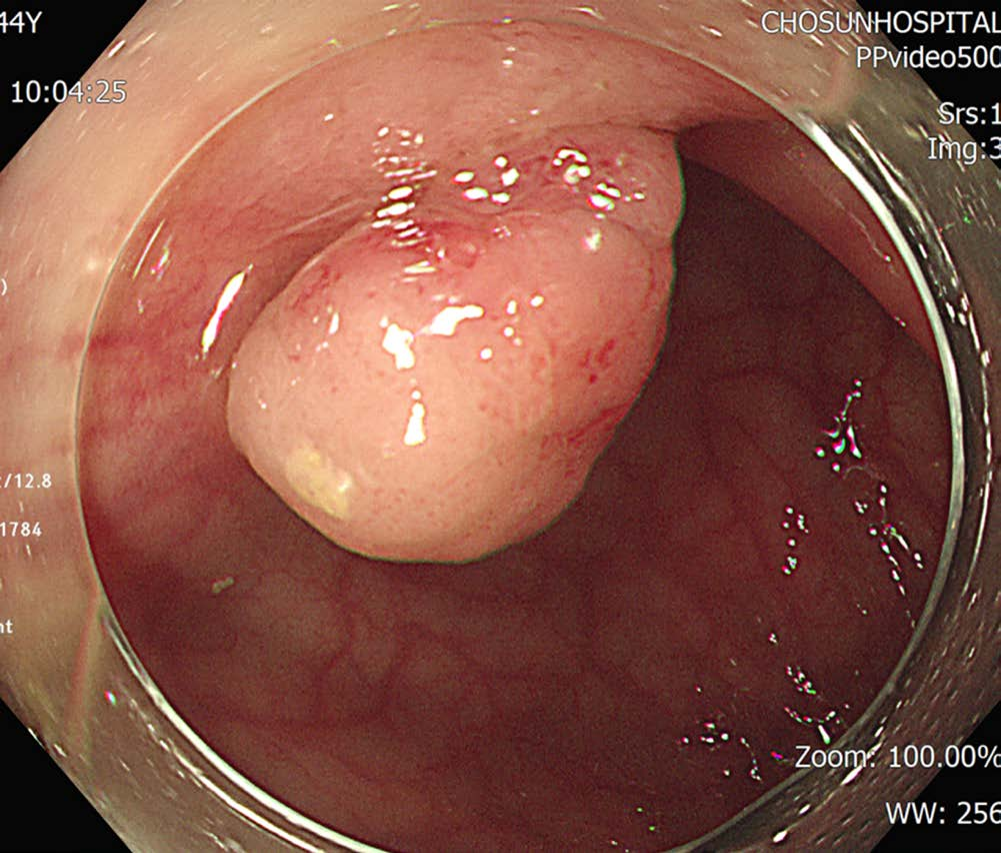
Endoscopic finding. Approximately 1 cm-sized fungating polyp at the ileocecal valve.

A 44-year-old man sought surgical intervention to treat severe symptoms of longstanding hemorrhoids. Multiple hemorrhoids with severe anal bleeding were observed in rectoscopy findings at the time of the outpatient visit, and the severity was grade III at the 3 and 7 o’ clock positions. Blood tests revealed no abnormal findings other than a hemoglobin of 7.6 g/dL. The patient had difficulty in digestion and persistent pain in the right lower abdomen, which led to additional colonoscopy. Polyps < 0.5 cm were observed in the ascending colon, descending colon, and sigmoid colon, all of which were subjected to polypectomy. The fungating polyp of the ileocecal valve (ICV) was approximately 1 cm in size, and the position of polyp also changed from time to time according to the bowel movement (Fig. 4). Therefore, EMR was performed later. After 20 days, the patient was hospitalized, and the ICV polyp was removed using underwater EMR, followed by hemorrhoidectomy.

**Figure 5.**
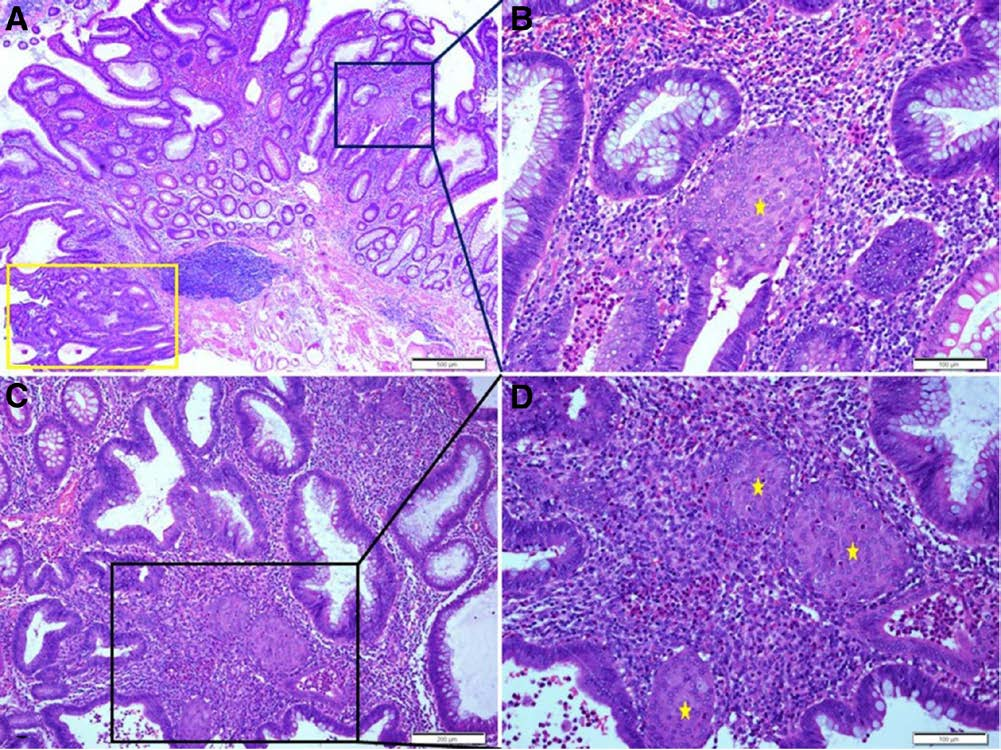
Histopathologic findings of the endoscopic mucosal resection. High grade tubular adenoma (yellow box) in the background of low-grade tubular adenoma is identified (A). Higher power view of the black boxed area of (A) shows low-grade adenoma with squamoid morules (asterisk, B). Another focus of low-grade adenoma with squamoid morules shows inflammatory cells infiltration around the squamoid morules (C). Higher power view of the black boxed area of (C) shows low-grade adenoma with squamoid morules (asterisks; D). Some lining cells of the glands are damaged and dropped, and inflammatory cells gather in the glandular lumens (B, D). Hematoxylin and eosin staining. Scale bars measure, (A) 500 µm, (B, D) 100 µm, and (C) 200 µm.

The histopathological findings of EMR were observed in one area with a high-grade dysplasia on a low-grade tubular adenoma background. The SM were scattered and observed in areas of low-grade dysplasia within the adenoma and were not seen in the focus of high-grade dysplasia. Severe infiltration of inflammatory cells around the SM was observed. In some glands, the lining cells of the glands were damaged and dropped, and inflammatory cells appeared gathered in the glandular lumens (Fig. 5).

**Figure 6.**
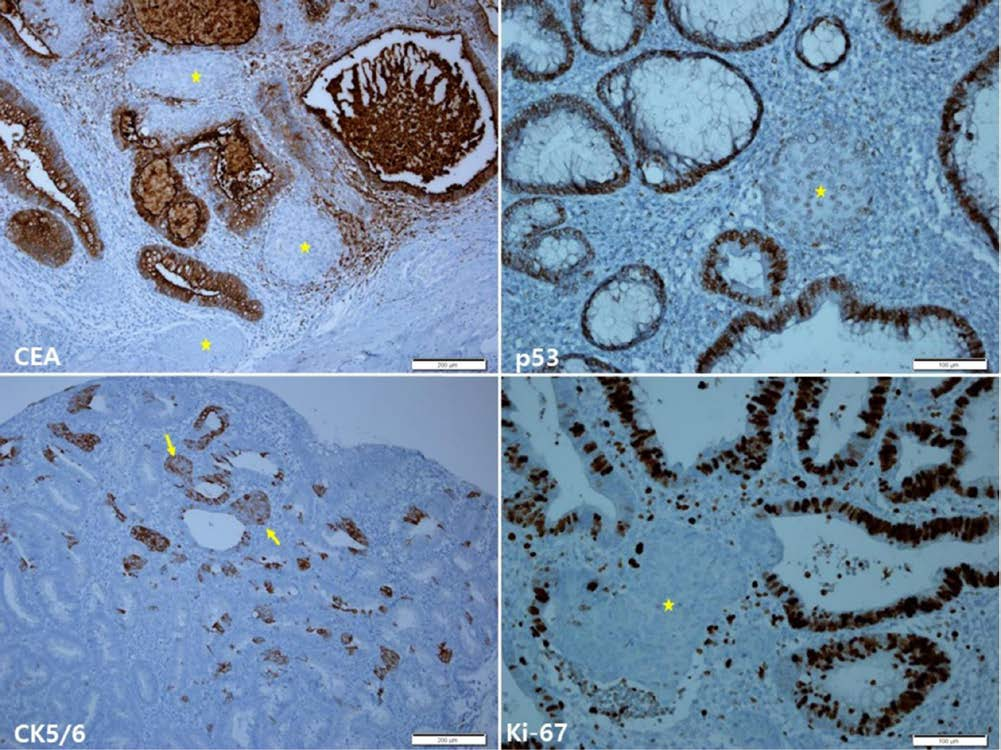
Immunohistochemical findings of the endoscopic mucosal resection. Adenoma shows positive expression of CEA, but the squamoid morules (asterisks) show negative expression of CEA (upper left). P53 protein staining shows diffuse strong expression in the adenoma, but weakly scattered expression in the squamoid morules (asterisk; upper right). CK5/6 staining shows positive expression in the squamoid morules (arrows) and some non-squamoid glandular epithelia (lower left). Ki-67 staining shows diffuse positive expression in the adenoma, but negative expression in the squamoid morules (asterisk; lower right). Scale bars measure, CEA and CK5/6: 200 µm and p53 and Ki-67: 100 µm. CEA = carcinoembryonic antigen.

The immunohistochemical findings of the EMR were similar to those in Case 1. The SM site was diffuse, strongly positive for CK5/6 and negative for p53 and Ki-67 staining. In addition, CK5/6 was observed in groups or scattered positive cells in areas that were not recognized as SM in hematoxylin and eosin (H&E) staining (Fig. 6).

No signs of recurrence were observed in colonoscopy performed 12 months after EMR.

## 3. Discussion

SM is a rare finding in colorectal adenoma.^[1–3]^ After the term morule was first used to describe squamous metaplasia of colorectal adenoma,^[5]^ it was argued that morule and squamous metaplasia should be used separately.^[6]^ However, the morphological changes associated with colorectal adenomas include squamous metaplasia, squamoid differentiation, and SM.^[1–3,5]^ It would be meaningless, in terms of the diagnosis and treatment of patients, to distinguish whether the findings seen in each case were squamous metaplasia or SM through ultrastructural analysis or immunohistochemical study. However, based on the immunohistochemical findings of the 2 cases currently reported, the term SM will be used.

However, the pathogenesis of SM in colorectal adenomas remains unclear.^[2,10]^ Polyps may vary depending on the type, but they are expected to always face mechanical trauma or irritation, chronic inflammation, and ischemia, especially pedunculated polyps, because they protrude into the lumen. This continuous irritation may cause metaplasia, and the intestinal mucosal cells are most likely converted into squamous cells.^[1,2]^ In the present case, various degrees of inflammatory cell infiltration were observed around the SM. In some glands, attenuation and denudation of the lining epithelia were observed with inflammatory cell infiltration. In Case 1, these findings were observed with distention of the glandular secretion. The findings in this case are presumed to support a pathogenesis that is rarely accompanied by SM in adenomas.

In addition, as a result of CK5/6 staining performed in these cases, positive findings were observed as individual cells or cell groups not only in the SM site, where H&E staining was recognized, but also in the surrounding adenomatous glandular lining cells, where H&E staining was not recognized as SM. This can be interpreted as having some cells that are not seen as SM by H&E staining undergoing squamous differentiation in terms of immunophenotype. In addition, the fact that the SM site was carcinoembryonic antigen-negative is considered a supporting finding.

The SM site, which is almost negative for p53 protein and has a low Ki-67 proliferation index, was in contrast to the pattern observed in the surrounding adenoma, which suggests that SM is a metaplastic change and not a dysplastic focus.

SM observed in colorectal adenoma occurs regardless of the location (surface, middle layer, or base) where the pseudoinvasion occurs. However, it was not detected in the residual normal glandular epithelium. In addition, in these cases, the SM site was negative for neuroendocrine markers such as synaptophysin, chromogranin A, CD56, and insulinoma-associated protein 1, but other studies have reported cases that are positive for neuroendocrine markers.^[9,10]^ These findings support the hypothesis that SM is caused by abnormal differentiation of the adenomatous epithelium or multipotent reserve cells beneath the adenomatous epithelium, rather than chronic irritation.^[9]^

According to the reported literatures,^[9,13]^ the incidence of colorectal adenoma with SM is approximately 0.4%, with an average age of 63 years and a male-to-female ratio of men and women (2:1). This can occur in the entire large intestine; however, the sigmoid colon is the most common, followed by the ascending colon and rectum at the same frequency, and is rare in other areas. Pedunculated polyps were more common than sessile polyps at a ratio of 6.5:1. The polyps were relatively large with an average of 2.3 cm. The histological subtypes of the adenomas were as follows: villotubular adenoma (61%), tubular adenoma (29%), and villous adenoma (11%). The ratio of low-grade dysplasia to high-grade dysplasia was 1.9:1, which is more common in low-grade dysplasia; therefore, it is not considered to be a finding that occurs as the dysplasia is more severe.

The patients reported in this study were relatively young (44 and 57), with ICV and descending colon, 1 and 3.2 cm, and fungating and pedunculated polyps, respectively, showing some differences from previous reports.

Submucosal pseudoinvasion of colorectal adenoma was first described by Muto et al^[7]^ with an incidence is 2% to 3% in polypectomy cases.^[9]^ It is most common in the sigmoid colon and occurs mostly (>90%) in polyps measuring > 1 cm in size. This needs to be differentiated from invasive cancer and is considered a pseudoinvasion if the glands are clearly demarcated from the surrounding stroma and composed of similar structures and cells without cytological atypia more severe than the gland observed in the head of the polyp.^[9,14–16]^

In addition, mucus retention or bleeding can lead to cystic dilatation of the submucosal glands, suggesting pseudoinvasion rather than invasive cancer if hemosiderin pigments are found or loose stroma is observed instead of desmoplasia.^[8–10]^ In Case 1, the appropriate findings were confirmed and judged to be submucosal pseudoinvasion.

It is very rare to see SM and submucosal pseudoinvasion simultaneously in colorectal adenomas; therefore, 10 cases have been reported, including Case 1.^[2,9–12]^ In this case, the average age of 58 years and the male-to-female ratio of 8:1 occurred mainly in men, common in the order of sigmoid, ascending, and descending colon. The ratio of pedunculated polyps and sessile polyps was 8:1, mainly found in pedunculated polyps. Histological subtypes included villotubular adenomas (5) and tubular adenomas (4) (Table 1).

**Table 1:** Clinicopathologic analysis of the colorectal adenomas with submucosal pseudoinvasion and squamoid morules.

Mean age (yr.)	Sex	Site	Gross type	Histologic type	Size (cm)
58.2 (44–73)	M: 8 F: 1	SC: 5 AC: 3 DC: 2	PP: 8 SP: 1	TVA (focal HG): 3 TA (LG): 3 TVA (LG): 2 TA (focal HG): 1	2.2 (1.0–3.5)
Total	9*	10	9*	9*	

AC = ascending colon, DC = descending colon, HG = high grade, LG = low grade, PP = pedunculated polyp, SC = sigmoid colon, SP = sessile polyp, TA = tubular adenoma, TVA = tubulovillous adenoma.

* Not reported in 1 case among the 10 cases.

Most colorectal adenomas with submucosal pseudoinvasion are pedunculated polyps, and the glands in the adenomatous pedicle may cause cystic dilation to form larger cysts, forming a thick and bulging stalk.^[9,12,17,18]^ On endoscopic ultrasound, these dilated cysts appear as hypoechoic masses in the pedicle, and when fluid or bleeding occurs in the cyst, hyperechoic areas are present. These findings do not indicate malignant submucosal invasion but are suggestive of pseudoinvasion of colorectal adenoma.^[17]^

In Case 1, cystic dilation and mucin spillage occurred in the pedicle, which seemed to be expressed in the form of a thick and bulging stalk.

Similar to submucosal pseudoinvasion of the mucosal glands, mucin spillage must be differentiated from invasive cancer.^[9]^ Mucin is produced by tumor cells in mucinous adenocarcinoma or adenocarcinoma with mucinous differentiation, resulting in mucus retention and cystic dilatation of neoplastic glands. If this continues to develop, mucin spills into the surrounding tissues.

In Case 1, mucin spillage was found at the submucosal pseudoinvasion site. In this case, no epithelial cells or cells with cytological atypia were observed in the mucin pool. However, a few inflammatory cells were observed in the mucin pool, and infiltration of inflammatory cells was observed. These findings are thought to reflect the physical and immunological responses to mucin outside the lumen.

To date, no cases of mucin spillage in colorectal adenoma patients with submucosal pseudoinvasion and SM have been reported. However, based on a report by Shi et al,^[9]^ this is probably considered a possible case of mucin spillage.

Meanwhile, findings of mucus retention in colorectal adenoma with SM and submucosal pseudoinvasion have been reported, but there were no cases of prominent mucin spillage, as in Case 1.

## 4. Conclusion

Colorectal adenoma with SM and submucosal pseudoinvasion is a rare finding, and 9 cases have been reported to date. We examined the pathogenesis of SM and submucosal pseudoinvasion through a literature review and examined the important differences between invasive cancers. In addition, in one case, in addition to the SM and submucosal pseudoinvasion findings, clear mucin spillage findings have not yet been reported; therefore, we report this in light of its rarity.

## Author contributions

**Conceptualization:** Sung-Chul Lim.

**Data curation:** Sung-Chul Lim.

**Formal analysis:** Song Iy Han.

**Funding acquisition:** Sung-Chul Lim.

**Investigation:** Sung-Chul Lim.

**Methodology:** Sung-Chul Lim.

**Supervision:** Sung-Chul Lim.

**Validation:** Song Iy Han.

**Visualization:** Song Iy Han.

**Writing – original draft:** Song Iy Han.

**Writing – review & editing:** Sung-Chul Lim.
